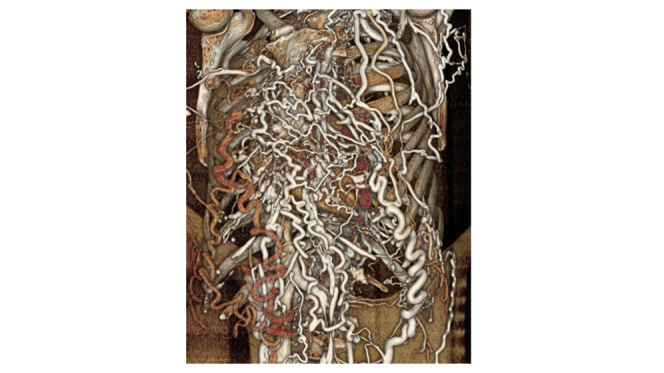# Extensive subcutaneous venous collateral network compensating for near-occlusive superior vena cava stenosis

**DOI:** 10.1016/j.jvsv.2026.102496

**Published:** 2026-04-02

**Authors:** Emma Morisot, Mickaël Ohana, Elena-Mihaela Cordeanu, Dominique Stephan

**Affiliations:** a Department of Hypertension and Vascular Diseases, University Hospital of Strasbourg, Strasbourg, France; b Department of Radiology, University Hospital of Strasbourg, Strasbourg, France

An 84-year-old woman with a history of bilateral breast cancer (2004, recurrence 2007, since in remission) treated by left radical mastectomy and right partial mastectomy, chylothorax managed by thoracic duct ligation and pleural talc pleurodesis, and atrial fibrillation, was referred by her general practitioner for suspicion of a superior vena cava syndrome. Physical examination revealed mild facial plethora, jugular venous distention, and a hepatojugular reflux, but no upper extremity edema, cyanosis, or dyspnea. A lateral view of the trunk (*A*) showed a striking network of tortuous, dilated subcutaneous veins extending from the anterior and lateral chest wall down to the lower abdomen, with a clearly caudal direction of flow. The left mastectomy scar was also visible. Computed tomography angiography revealed a chronic, near-occlusive stenosis of the superior vena cava (SVC) proximal to the azygos arch, likely secondary to post-therapeutic sequelae from breast cancer treatment. Three-dimensional volume-rendered reconstruction (*B*/Cover) demonstrated two compensatory drainage pathways: paravertebral collaterals draining into the azygos system, and innumerable subcutaneous collaterals coursing along the anterior thoracoabdominal wall, ultimately draining into the bilateral common iliac veins. The inferior vena cava appeared slender but patent. Duplex ultrasound examination confirmed collateral patency with caudally directed flow and no thrombosis. Echocardiography showed preserved left ventricular function, moderate precapillary pulmonary hypertension, and mild right ventricular dilatation. Given the chronic, hemodynamically compensated nature of the SVC obstruction, conservative management was adopted.

This case illustrates that slowly progressive SVC obstruction may not result in overt SVC syndrome when the venous system has sufficient time to develop alternative drainage pathways.^1^^,^^2^ Here, the predominant collateral route exploited the anterior thoracoabdominal subcutaneous veins, rerouting the entire upper body venous return toward the iliac venous confluence, a pathway well-described anatomically but rarely demonstrated with such dramatic clinical and imaging correlation.^3^^,^^4^ Clinicians should recognize that extensive chest wall collateral veins warrant investigation for SVC pathology, even in the absence of classical SVC syndrome.^5^ The patient consented to the publication of this report and accompanying images.

**Figure:**
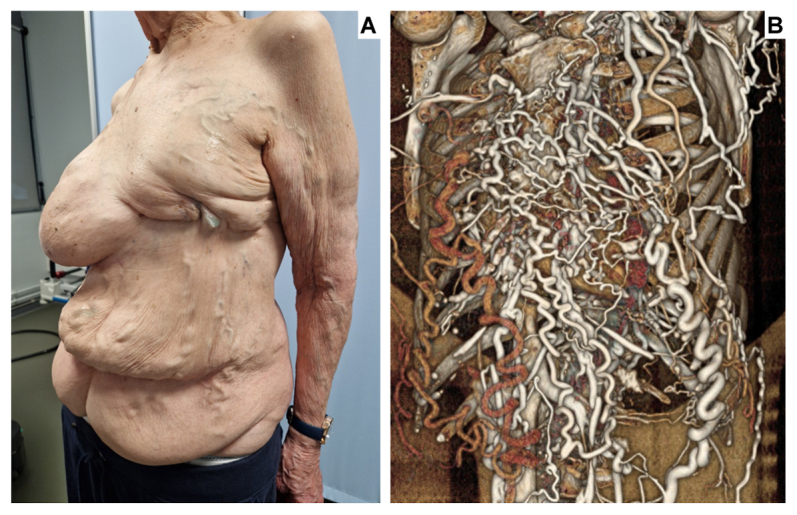

## Funding

None.

## Disclosures

None.

## Figures and Tables

**Cover image:**